# Chemotherapy re-use versus anti-angiogenic monotherapy as the third-line treatment of patients with metastatic colorectal cancer: a real-world cohort study

**DOI:** 10.1186/s12885-024-12072-5

**Published:** 2024-03-05

**Authors:** Jingjing Duan, Lila Zhu, Yinghui Shi, Weixue Wang, Tongtong Wang, Tao Ning, Le Zhang, Ming Bai, Hongli Li, Rui Liu, Shaohua Ge, Xia Wang, Yuchong Yang, Zhi Ji, Feixue Wang, Yansha Sun, Yi Ba, Ting Deng

**Affiliations:** Department of GI Medical Oncology, Tianjin’s Clinical Research Center for Cancer, Tianjin Key Laboratory of Digestive Cancer, Key Laboratory of Cancer Prevention and Therapy, Tianjin Medical University Cancer Institute and Hospital, National Clinical Research Center for Cancer, Huan huxi Road, 300060 Tianjin, Tianjin, China

**Keywords:** Colorectal cancer, Third-line therapy, Chemotherapy, Real-world evidence

## Abstract

**Background:**

There are various recommendations for third-line treatment in mCRC, however, there is no consensus on who is more suitable for particular strategy. Chemotherapy re-use in third-line setting is a common option in clinical practice. This study aimed to investigate the efficacy of third-line chemotherapy re-use by the comparison with that of anti-angiogenic monotherapy, and further find the population more suitable for third-line chemotherapy.

**Methods:**

Using electronic medical records of patients with mCRC, a retrospective cohort study was conducted. A total of 143 patients receiving chemotherapy and 40 patients receiving anti-angiogenic monotherapy in third-line setting as control group were retrospectively collected. Baseline characteristics were analyzed using the χ² test or the Fisher’s exact test. ROC curve and surv_cutpoint function of ‘survminer’ package in R software were used to calculate the cut-off value. Survival curves were plotted with the Kaplan-Meier method and were compared using the log-rank test. The Cox proportional hazard regression model was used to analyze the potential risk factors.

**Results:**

A total of 143 patients receiving chemotherapy and 40 patients receiving anti-angiogenic monotherapy in third-line setting were retrospectively collected. Chemotherapy rechallenge was recorded in 93 patients (93/143, 65.0%), and the remaining patients chose new chemotherapeutic drugs that had not been previously used, including irinotecan-based (22/50), oxaliplatin-based (9/50), raltitrexed (9/50), gemcitabine (5/50) and other agents (5/50). The ORR and DCR of third-line chemotherapy reached 8.8%, 61.3%, respectively (anti-angiogenic monotherapy group: ORR 2.6%, DCR 47.4%). The mPFS and mOS of patients receiving chemotherapy were 4.9 and 12.0 m, respectively (anti-angiogenic monotherapy group: mPFS 2.7 m, mOS 5.2 m). Subgroup analyses found that patients with RAS/RAF mutation, longer PFS (greater than 10.6 m) in front-line treatment or larger tumor burden had better prognosis with third-line chemotherapy rather than anti-angiogenic monotherapy.

**Conclusions:**

Third-line chemotherapy re-use was effective in mCRC. Those with more aggressive characteristics (RAS/RAF mutant, larger tumor burden) or better efficacy of previous chemotherapy (longer PFS) were more appropriate for third-line chemotherapy, rather than anti-angiogenic monotherapy.

**Supplementary Information:**

The online version contains supplementary material available at 10.1186/s12885-024-12072-5.

## Background

Colorectal cancer (CRC) is a commonly diagnosed malignant tumor with the second highest mortality worldwide [1]. In China, the incidence of CRC is increasing, and a large proportion of patients are confirmed to be metastatic CRC (mCRC) cases at the time of initial diagnosis. The development of medical therapies in mCRC patients has not been so transformative as in other malignancies, mainly because the number of patients who can benefit from targeted therapy or immunotherapy is relatively limited [2]. Thus, chemotherapy remains the backbone treatment for mCRC. Standard first-line or second-line chemotherapy regimens include the combination of fluorouracil, folic acid and oxaliplatin/irinotecan (FOLFOX/FOLFIRI) or other oxaliplatin/irinotecan-based regimens. Although the clinical efficacy has been improved by the combination of chemotherapy with anti-angiogenic drugs [3] or anti-EGFR agents [4], the 5-year survival rate of mCRC remains unsatisfied.

For the third-line therapy of mCRC, guidelines recommend regorafenib [5, 6], trifluridine/tipiracil (TAS-102) [7], and rechallenge with anti-EGFR treatment in patients with RAS wild-type disease. Since the randomized phase III SUNLIGHT trial showed that treatment with TAS-102 plus bevacizumab resulted in longer overall survival than TAS-102 alone, this combination was also recommended in the third-line therapy [8]. However, in clinical practice, one option is to reintroduction or rechallenge chemotherapy in their third-line setting. Unlike reintroduction of therapy occurs in situations where there is no resistance, rechallenge indicates the administration of drugs in which the tumor has developed resistance [9]. Although the mechanism supporting rechallenge has not been fully understood [10], it may be beneficial for patients to receive rechallenge strategy under certain circumstances. However, the efficacy of this strategy in the third-line setting is not clearly established.

The most widely known anti-tumor drug to use rechallenge strategy is cetuximab. Cetuximab preferably in combination with irinotecan, is alternative third-line choice in KRAS/NRAS/BRAF wild-type patients. In a phase II, single arm trial of mCRC patients with irinotecan and cetuximab resistance, the ORR of cetuximab plus irinotecan reached 54% [11]. More studies were carried out subsequently to further confirm the efficacy of the biomarker guided cetuximab rechallenge therapy [12, 13]. The randomized phase II VELO trial further verified that the addition of panitumumab to TAS-102 as anti-EGFR rechallenge therapy significantly improved progression-free survival (PFS) as compared to TAS-102 in third-line therapy in patients with RAS wild-type (WT) mCRC [14, 15]. As for the efficacy of chemotherapy re-use in third-line setting, the evidence is limited and there have been no prospective randomized trails by far. Several studies explored the efficacy of oxaliplatin re-use [16, 17] or irinotecan re-use [18, 19], and the reported survival outcome was promising. However, whether it is comparable with the standard third-line anti-angiogenic monotherapy remains largely unknown.

This study aimed to investigate the efficacy of third-line chemotherapy re-use by the comparation with anti-angiogenic monotherapy in mCRC, and further find the population more suitable for third-line chemotherapy through subgroup analyses.

## Patients and methods

### Study design and patients

This retrospective study aimed to assess the efficacy of third-line chemotherapy for mCRC. Patients were carefully screened based on the following inclusion and exclusion criteria. The inclusion criteria were as follows: pathologically confirmed colorectal cancer; metastatic or unresectable colorectal cancer; third-line treatment was recorded as chemotherapy with or without targeted drugs, or anti-angiogenic monotherapy (regorafenib, fruquintinib or anlotinib); available follow-up. Patients were removed from the study if one of the following events occurred: had other malignant tumors during the baseline period; the occurrence of a second primary cancer during the follow-up period.

Consecutive patients with mCRC meeting the above criteria from the Tianjin Medical University Cancer Institute and Hospital between January 2013 and December 2020 were selected. Demographic data, clinicopathological information and treatment details are collected from electronic medical records.

Due to the retrospective nature of this study, the ethical approval and informed consent requirements from each patient have been waived according to the regulations of the Tianjin Medical University Cancer Institute and Hospital Ethics Committee.

### Treatment

The date on which the third-line treatment started was defined as the index date. The follow-up period started on the date of third-line treatment initiation and ended on the date of data cut-off, the date of the last visit or the date of death. Baseline clinical characteristics were evaluated before or at the index date of the third-line treatment. The drugs and cycles of each line treatment were recorded in detail.

### Outcomes measures

Tumor response results according to the RECIST criteria were recorded as complete response (CR), partial response (PR), stable disease (SD) and disease progression (PD). When the time of death or progression cannot be determined or the patient was still alive, the date of the last follow-up was recorded as censored data in survival analyses. PFS was calculated from the date of treatment initiation to the date of diagnosis of progression or death (whichever occurred first) or the last follow-up (for the censored patients). PFS1, PFS2 and PFS3 referred to PFS of first-line, second-line and third-line therapy, respectively. The period from the start of treatment to the date of death from any cause or the last contact (for the censored patients) was recorded as overall survival (OS), and OS3 was considered as the time from the beginning of the third-line treatment to death or the last follow-up.

### Statistical analysis

Continuous variables were summarized by the mean values and standard deviation, which were compared with the students’ t test when the normality and homoscedasticity are satisfied. Otherwise, non-parametric test was used in non-normal data. Categorical variables were recorded as percentages and analyzed using the χ² test or the Fisher’s exact test. ROC curve and surv_cutpoint function of ‘survminer’ package in R software were used to calculate the cut-off value. Survival curves were plotted with the Kaplan-Meier method and were compared using the log-rank test. In the case of the proportionality of risks, the Cox proportional hazard regression model was used to analyze the potential risk factors. Statistical analyses in this study were conducted with the IBM SPSS Statistics, Version 20.0. A *P* value of < 0.05 was considered as statistically significant. All statistical tests were two-tailed. Lastly, a post hoc (a posteriori) power analysis was conducted to verify the reliability of the results with the PASS software.

## Results

### Baseline clinicopathological characteristics

Between January 2013 and December 2020, 183 patients with mCRC who met the inclusion and exclusion criteria from the Tianjin Medical University Cancer Institute and Hospital were selected. Among them, 143 patients received chemotherapy ± targeted drugs, and the remaining 40 patients were given anti-angiogenic monotherapy. The baseline disease characteristics at the index date of third-line therapy were listed in Table 1.

**Table 1:** Baseline disease characteristics and treatment patterns (baseline characteristics were assessed at the index date of third-line treatment)

Characteristics	Third-line chemotherapy ± targeted drugs (*n* = 143)		Third-line anti-angiogenic drugs (*n* = 40)			*P*
**Disease characteristics**						
Age (years)						0.462^§^
Mean (range)	58 (24–80)	57 (29–83)				
Gender						0.435
Male	78		24			
Female	65		15			
Location of primary tumor						0.465^*^
Ascending colon	34		6			
Transverse colon	10		1			
Descending colon	10		1			
Sigmoid colon	57		20			
Rectum	28		10			
Unknown	4		2			
Pathologic differentiation						0.571^a^
Adenocarcinoma	99		31			0.271^b^
Well differentiated	7		2			
Moderately differentiated	71		18			
Poorly differentiated	21		11			
Mucinous adenocarcinoma	12		2			
Unknown	32		7			
Stage at initial diagnosis						0.579^*^
I	3		0			
II	7		0			
III	41		15			
IV	82		23			
Unknown	10		2			
Resection of primary tumor						0.775
Radical resection	61		15			
Palliative resection	56		16			
None	26		9			
RAS/RAF status^#^						0.182
Wild-type	44		8			
Mutant-type	99		32			
Number of metastatic organs at third-line treatment						0.185
1	73		24			
2	28		10			
≥ 3	42		6			
Liver-limited metastases at third-line treatment						0.584
Yes	47		15			
No	96		25			
***Adjuvant chemotherapy patterns***						
Adjuvant chemotherapy						0.732
Yes	96		28			
No	47		12			
Cycles of adjuvant chemotherapy						0.673^£^
Median (range)	6 (1–12)	6 (3–12)				
***First-line treatment patterns***						
Treatment						0.830
Chemotherapy alone	93		20			
Chemotherapy plus targeted drugs	46		9			
Targeted drugs						0.894
Anti-VEGF drugs	31		7			
Anti-EGFR drugs	14		2			
Cycles						0.228^£^
Median (range)	8 (1–18)	6 (2–18)				
***Second-line treatment patterns***						
Treatment						0.348^*^
Chemotherapy alone	69		23			
Targeted drugs alone	4		2			
Chemotherapy plus targeted drugs	67		13			
Immunotherapy combination	3		0			
Targeted drugs						0.282
Anti-VEGF drugs	63		15			
Anti-EGFR drugs	10		0			
Cycles						0.814^£^
Median (range)	6 (1–22)	6 (1–12)				
***Third-line treatment patterns***						
Previous use of targeted drugs						0.145
Anti-VEGF + Anti-EGFR	7			2		
Anti-VEGF in 1st- or 2nd-line treatment	43			8		
Anti-VEGF in 1st- and 2nd-line treatment	22			6		
Anti-EGFR in 1st- or 2nd-line treatment	14			0		
None	57			22		
Chemotherapy						
Chemotherapy re-challenge	93		-			
New chemotherapy regimens	50		-			
Targeted drugs						-
Targeted drugs re-challenge	49		0			
New targeted drugs	37		40			
Anti-angiogenic drugs						-
Yes	62		40			
No	81		0			
Cycles						-
Median (range)	4 (1–20)		2 (1–12)			
***Later-line treatment***						0.558
Yes	50		12			
No	93		28			

§: t test; *: Fisher exact probability test; £: non-parametric test

*a: Test for different degrees of differentiation;*

*b: Test for different pathological type;*

*#: RAS/RAF status was determined in initial tumor diagnosis*

The median age of mCRC patients at third-line treatment initiation was 58 years (range: 20–84 years). As expected, more male patients were included (103/183, 56.3%). Sigmoid colon was the most common tumor location (77/183, 42.1%), followed by ascending colon (40/183, 21.9%) and rectum (38/183, 20.8%). All tumors in our cohort were defined as pMMR, and 28.4% of cases were RAS/RAF-wild type. At the time of initial diagnosis, most patients had been diagnosed with stage IV disease (105/183, 57.4%). Nearly half of patients (76/183, 41.5%) were given radical resection at the initial treatment, and 77.6% (59/76) of them received adjuvant chemotherapy. The most frequently used adjuvant chemotherapy regimen (51/59, 86.4%) was the combination of oxaliplatin with fluoropyrimidine, and the rest were given fluorouracil monotherapy (3/59, 5.1%) or others (5/59, 8.5%).

### First-line and second-line treatment patterns

Since there were 15 patients with incomplete first-line treatment information, we collected details from the remaining 168 patients (91.3%, Table 1). Among these patients, only 32.7% of patients were given chemotherapy plus targeted drugs (55/168), whereas more than two-thirds of patients chose chemotherapy alone (113/168, 67.3%) due to economic constraints or concerns about adverse events. As shown in Fig.S1A, the most frequently used chemotherapy regimens were FOLFOX and other oxaliplatin-based regimens (68.9%), followed by FOLFIRI and other irinotecan-based chemotherapy (18.6%). In the use of targeted drugs, more than 20% of patients chose bevacizumab, whereas less than 10% of patients received anti-EGFR therapy. The median PFS1 was 8.0 m (range: 1.3-41.3 m) and 9.4 m (range: 1.5-44.0 m) in patients receiving chemotherapy alone and those receiving the combination of chemotherapy and targeted drugs, respectively.

In 181 patients available for second-line therapy details, chemotherapy with or without targeted drugs remained the dominated choice (172/181, 95.0%). Due to relatively poor physical status or other personal choice, 3.3% (6/181) and 1.7% (3/181) of patients were prescribed with targeted therapy alone or immunotherapy, respectively. Different from the first-line schemes, FOLFIRI and other irinotecan-based chemotherapy were the most frequently used regimen (68.3%, Fig.S1B), whereas only 21.9% of patients were given oxaliplatin-based regimens in their second-line setting. In the use of targeted drugs, similar to the first-line data, bevacizumab was still the most commonly used drug. The median PFS2 was 6.0 m (range: 1.0-28.0 m), 5.4 m (range: 1.0-24.0 m) and 8.0 m (range: 1.0-28.0 m) in all patients, patients receiving chemotherapy alone and those with chemotherapy plus targeted drugs, respectively.

In regard to the overall use of targeted drugs in front-line treatment, more than half of patients had used targeted drugs (102/181, 56.4%, Table 1) in our cohort. Among them, 93 patients had received a single type of targeted agents, and 15.4% (28/181) of them had used bevacizumab across lines. The remaining 9 patients were given sequential prescription of anti-EGFR and anti-VEGF drugs.

### The features of third-line chemotherapy

**Fig. 1:**
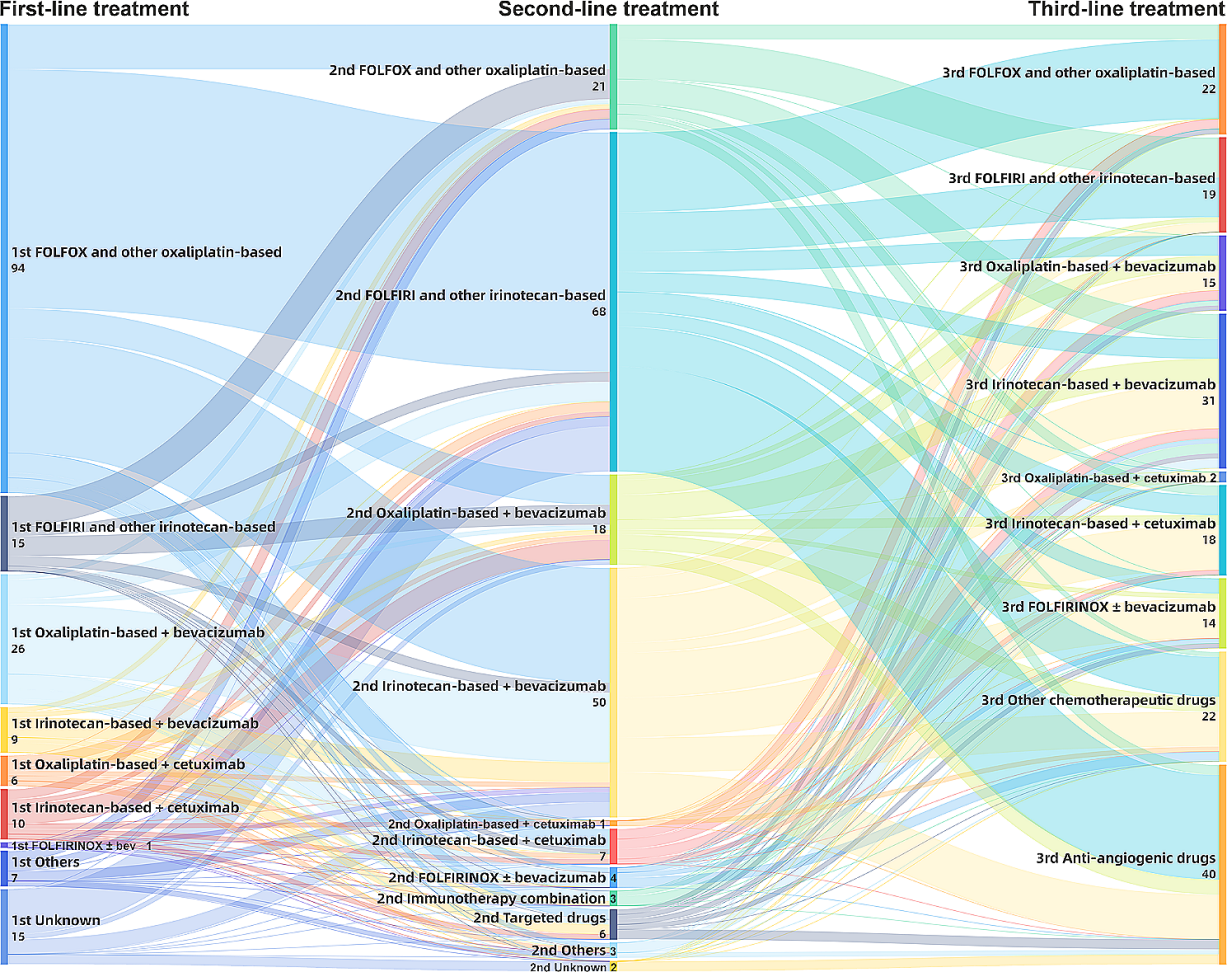
Treatment sequences of palliative chemotherapy for mCRC patients

At the initiation of third-line treatment, more than half of patients’ metastases were limited to a single organ (97/183, 53.0%). The most common metastatic sites involved the liver (63.4%), followed by lung (29.0%), peritoneum (15.8%), lymph nodes (10.9%), ovary (7.7%) and bone (4.9%) (Fig.S2A). As shown in Table 1 and Fig.S2B, 143 mCRC patients in our cohort were treated with chemotherapy in third-line setting. Among them, 86 patients chose the combination with targeted drugs, and 72.1% (62/86) of them were given bevacizumab. Chemotherapy re-challenge was recorded in 93 patients (93/143, 65.0%), and the remaining patients chose new chemotherapeutic drugs that had not been previously used, including irinotecan-based (22/50, 44.0%), oxaliplatin-based (9/50, 18.0%), raltitrexed (9/50, 18.0%), gemcitabine (5/50, 10.0%) and other agents (5/50, 10.0%). Similarly, targeted drugs rechallenge strategy including cetuximab and bevacizumab rechallenge was also prescribed in 49 patients. As shown in Fig. 1, regimens containing oxaliplatin, irinotecan, bevacizumab or cetuximab run through the first-line to the third-line treatment of mCRC. 84.6% (121/143) of patients still chose conventional treatment schemes, such as FOLFOX or FOLFIRI ± bevacizumab or cetuximab. As for later-line treatments, more than one-third of patients (62/183, 33.9%) had records, including chemotherapy, immunotherapy and clinical trials of new drugs.

### The efficacy of third-line chemotherapy

Since anti-angiogenic monotherapy has become the standard third-line treatment for mCRC, this study compared the efficacy of third-line chemotherapy with that of third-line anti-angiogenic drugs. As shown in Table 1, there was no difference in baseline characteristics and front-line treatment patterns between the two schemes. Tumor response assessment results were obtained in 175 (175/183, 95.6%) cases. The ORR and DCR reached 8.8% and 61.3% in patients receiving chemotherapy, respectively (Table 2). However, the ORR and DCR data only reached 2.6% and 47.4% in those with anti-angiogenic monotherapy, respectively.

**Table 2:** Response assessment of third-line treatment

Assessment	Chemotherapy ± targeted drugs				Anti-angiogenic drugs (No.)
	Total (No.)	Chemotherapy alone (No.)	Chemotherapy + targeted drugs (No.)		
PR	12	5	7		1
SD	72	23	49		17
PD	53	27	26		20
ORR	8.8%	9.1%	8.5%		2.6%
DCR	61.3%	50.9%	68.3%		47.4%

*CR: complete response, PR: partial response, SD: stable disease, PD: disease progression, ORR: overall response rate, DCR: disease control rate*

**Fig. 2:**
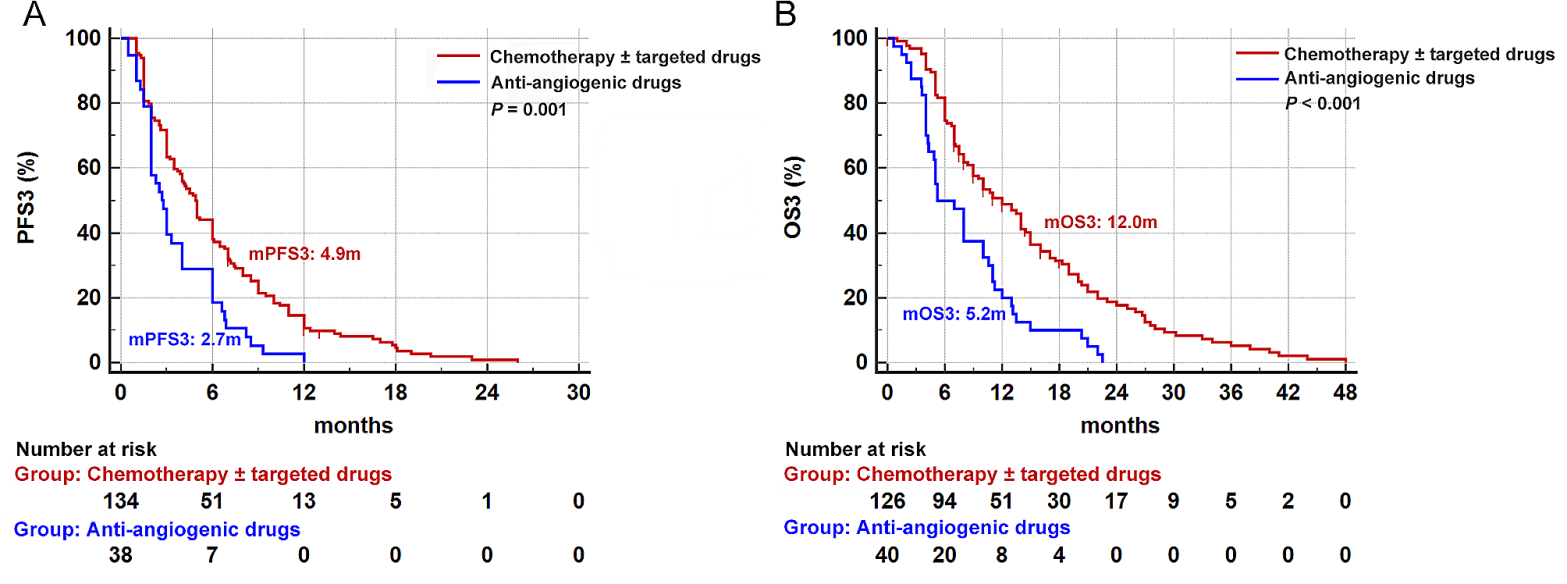
The survival curves of patients in different third-line treatment. **(A)** The progression-free survival curves of patients stratified by third-line treatment. **(B)** The overall survival curves of patients stratified by third-line treatment

The median follow-up time in our cohort was 9.0 months (range: 1.0–48.0 months). Disease progression and time of death were recorded in 169 (169/183, 92.3%) and 151 patients (151/183, 82.5%), respectively. The median PFS3 was 4.9 m in patients receiving chemotherapy ± targeted drugs, which was superior to that of those treated with anti-angiogenic monotherapy (2.7 m, *P* = 0.001, Fig. 2A). Likewise, the OS3 of patients receiving chemotherapy ± targeted drugs were also better than that of control group (12.0 m vs. 5.2 m, *P* < 0.001, Fig. 2B). However, whether patients selected chemotherapy rechallenge or new chemotherapeutic drugs did not affect survival (Fig. S3). Furthermore, due to the combination of targeted drugs in the chemotherapy group and the heterogeneity of tumor characteristics, we conducted subgroup analyses to explore the population more suitable for third-line chemotherapy.

### Patients suitable for third-line chemotherapy

#### RAS/RAF status

It should be noted that 80.0% (32/40) of patients in the anti-angiogenic monotherapy group were RAS/RAF mutant type, whereas the figure was 69.2% (99/143) in the chemotherapy group (Table 1). Since the biological features and prognosis are completely different between RAS/RAF wild type and RAS/RAF mutant tumors, it is not credible to compare the survival of the two groups in the whole population. Thus, we firstly conduct subgroup analysis based on different RAS/RAF status.

**Fig. 3:**
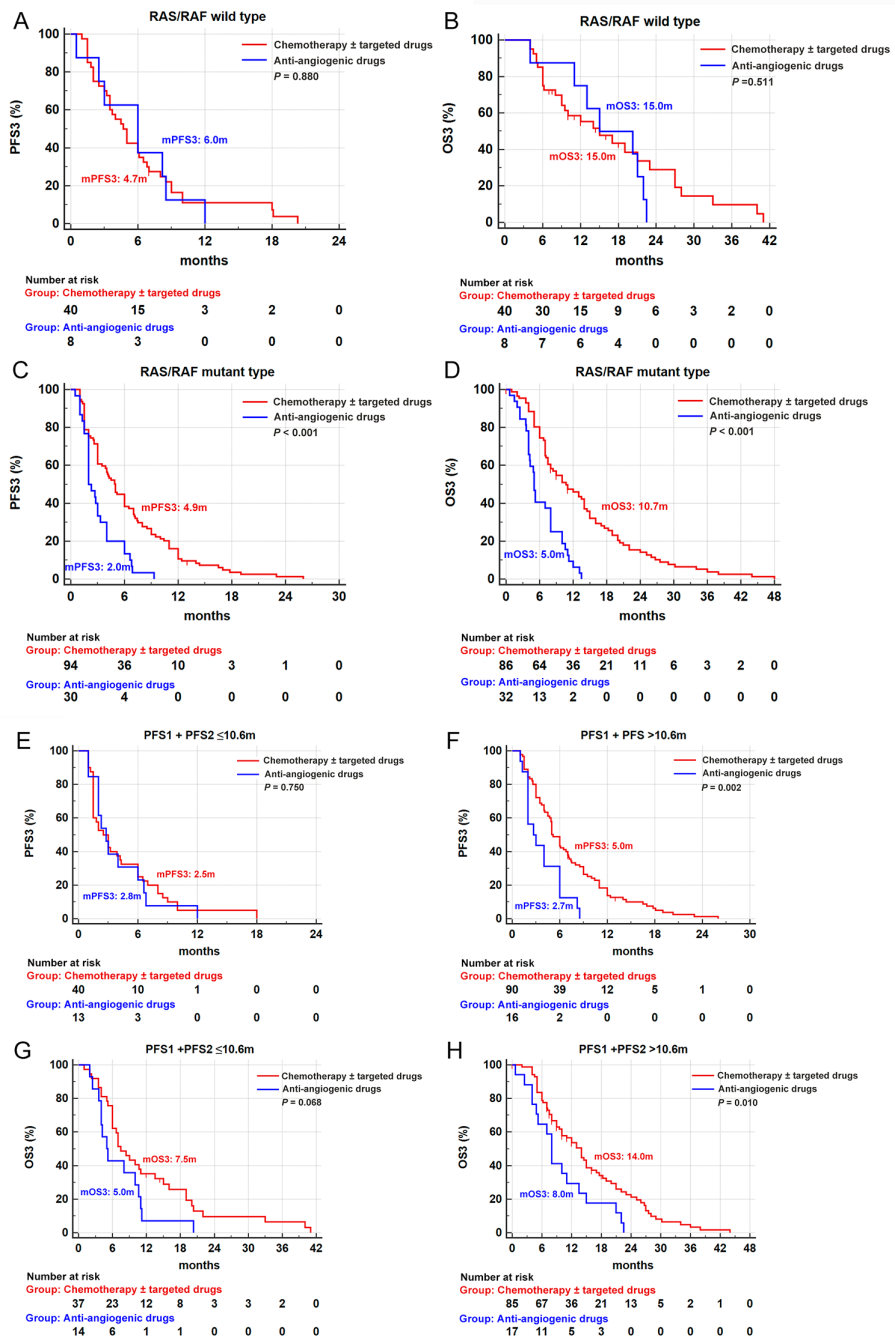
The survival curves of patients in different subgroups. **(A-B)** The progression-free survival curves stratified by RAS/RAF status. **(C-D)** The overall survival curves stratified by RAS/RAF status. **(E-F)** The progression-free survival curves stratified by the PFS of front-line treatment. **(G-H)** The overall survival curves stratified by the PFS of front-line treatment. PFS: progression-free survival. OS: overall survival

In RAS/RAF wild tumors of the chemotherapy group (*n* = 44), 9 patients, 13 patients and 22 patients were given chemotherapy alone (9/44, 20.5%), the combination of bevacizumab (13/44, 29.5%) and the combination of cetuximab (22/44, 50.0%), respectively. As shown in Fig. 3A-B, there was no difference in the survival of patients receiving third-line chemotherapy or anti-angiogenic monotherapy in the RAS/RAF wild-type population. Moreover, the combination of bevacizumab and cetuximab did not further increase the efficacy of third-line chemotherapy (Fig. S4A-B) in this subgroup.

In RAS/RAF mutant tumors of the chemotherapy group (*n* = 99), nearly half of patients were given chemotherapy alone (48/99, 48.5%), and the remaining patients chose the combination of bevacizumab (51/99, 51.5%). From the survival curve shown in Fig. S4C-D, whether or not bevacizumab was used in combination had no effect on the efficacy of third-line chemotherapy. Overall, the survival of patients in the chemotherapy group was superior to that of the anti-angiogenic monotherapy group (Fig. 3C-D), indicating that patients with RAS/RAF mutation seem to be more suitable to select chemotherapy at their third-line treatment.

### PFS of front-line treatment

In addition to RAS/RAF status, we assumed that if the PFS obtained from front-line treatment is relatively longer, the probability of benefit from the conventional chemotherapy scheme at the third-line will be greater. Therefore, the surv_cutpoint function of ‘survminer’ package in R software was used to find the cut-off value of PFS of front-line treatment, and the calculation showed that the cut-off value of PFS1 + PFS2 was 10.6 m. Besides, ROC curve was analyzed to verify the cut-off value. Since the reported PFS data of regorafenib is less than 4 months [5, 6], we assumed that patients receiving third-line chemotherapy with PFS greater than 4 months were the benefit population, which then acted as the outcome variable. As shown in Fig. S5, the PFS of front-line treatment had significant predictive ability (AUC:0.635, *P* = 0.009). According to the calculation result of Youden index, 10.7 months was considered as the cut-off value, which was consistent with that of surv_cutpoint function. Hence, we used the sum of PFS1 plus PFS2 as a stratification factor to further compare the efficacy of third-line chemotherapy scheme with the standard anti-angiogenic monotherapy.

As shown in the Fig. 3E-H, in the population whose PFS1 + PFS2 was greater than 10.6 m, the third-line choice of chemotherapy re-use had more survival advantages than anti-angiogenic monotherapy. However, in those with PFS1 + PFS2 equal to or less than 10.6 m, the survival of cases receiving third-line chemotherapy had no significant superiority to that of cases receiving anti-angiogenic monotherapy, demonstrating that the sum of PFS1 plus PFS2 could be regarded as a positive indicator for third-line chemotherapy.

### Other factors

**Fig. 4:**
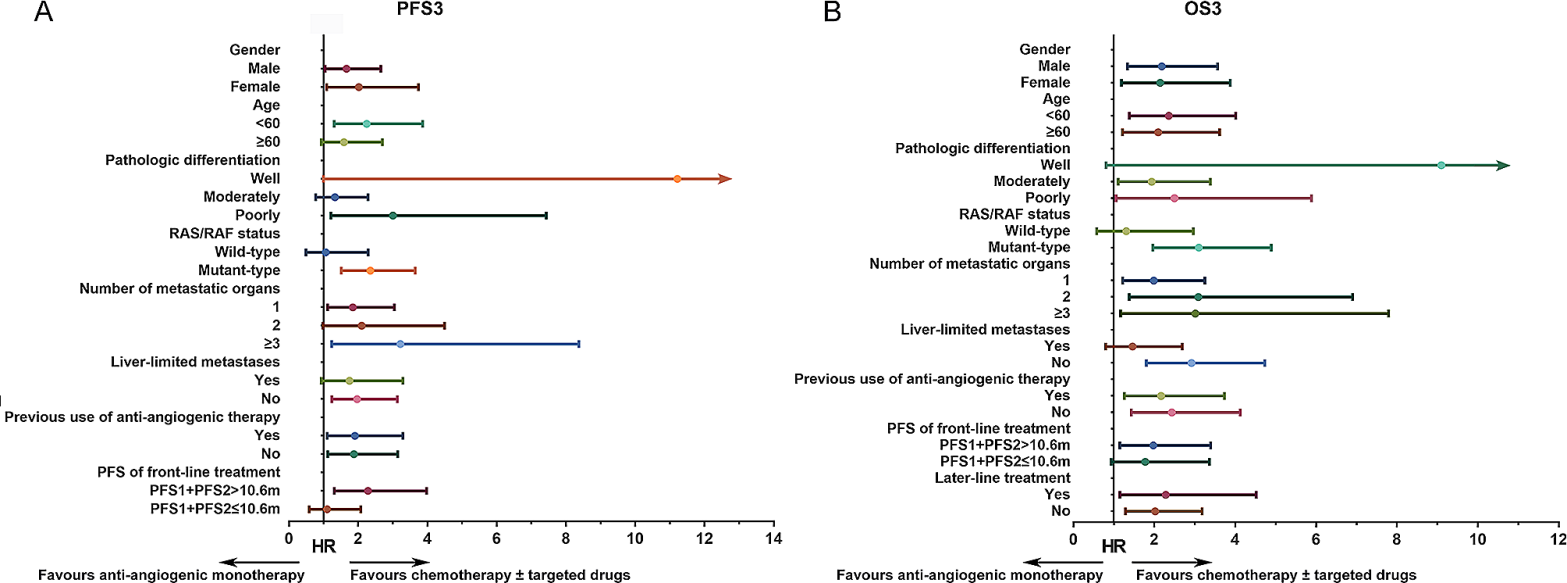
Subgroup analysis. We calculated HRs and CIs using the unstratified Cox regression model for the subgroup progression-free survival analysis **(A)** and overall survival analysis **(B)**. Error bars are 95% CIs. PFS: progression-free survival. OS: overall survival. HR: hazard ratio. CI: confidence interval

Subgroup analyses using the unstratified Cox regression model were performed and further validated the predictive value of RAS/RAF status and the PFS of front-line treatment (Fig. 4). Besides, for patients with larger tumor burden (tumor metastases were not only limited to the liver, or even involving more than or equal to three organs), it might be more suitable to choose powerful combination therapy such as chemotherapy combined with or without targeted therapy, rather than anti-angiogenic monotherapy.

### Analysis of prognostic factors

Finally, due to all data meeting the proportionality of risks, the Cox regression model was used to determine prognostic factors in the whole population. The univariate results showed that the third-line therapeutic regime, the longer PFS of front-line therapy were significantly associated with differential hazard for PFS3 (Table 3). After the multivariate analyses, the above two characteristics were found to be independent prognostic factors for PFS3. Similarly, the Cox model for OS3 demonstrated that the third-line therapeutic regime, RAS/RAF status, PFS of front-line therapy and whether or not there was later-line treatment could act as independent prognostic factors for OS3 after the multivariate adjustment.

**Table 3:** The Cox univariate and multivariate analyses of mCRC patients receiving third-line therapy

Factor	PFS3				OS3		
	*P*	HR	95%CI	*P*		HR	95%CI
**Cox univariate analyses**							
Gender (Male vs. Female)	0.097	0.766	0.559–1.049	0.367		0.858	0.616–1.196
Age (< 60 vs. ≥60)	0.596	0.921	0.679–1.249	0.500		0.895	0.647–1.237
Location of primary tumor							
Transverse colon vs. Ascending colon	0.532	1.243	0.628–2.460	0.246		0.648	0.312–1.347
Descending colon vs. Ascending colon	0.846	1.070	0.542–2.112	0.690		1.154	0.571–2.330
Sigmoid colon vs. Ascending colon	0.933	1.018	0.675–1.535	0.814		0.952	0.630–1.438
Rectum vs. Ascending colon	0.860	1.043	0.651–1.671	0.139		0.695	0.429–1.126
Pathologic differentiation							
Well vs. Poorly	0.628	0.811	0.349–1.889	0.779		0.886	0.382–2.058
Moderately vs. Poorly	0.336	1.238	0.801–1.916	0.363		0.808	0.510–1.279
**Resection of primary tumor**							
Palliative vs. Radical resection	0.867	1.029	0.733–1.446	0.064		1.403	0.981–2.007
**None vs. Radical resection**	**0.011**	**1.730**	**1.131–2.645**	**0.010**		**1.816**	**1.155–2.854**
**RAS/RAF status** **(Wild-type vs. Mutant-type)**	0.563	0.904	0.644–1.271	**0.011**		**0.615**	**0.424–0.893**
Number of metastatic organs at third-line treatment	0.456	1.074	0.890–1.296	0.281		1.121	0.911–1.381
**PFS of front-line treatment** **(PFS1 + PFS2 > 10.6 m vs. ≤10.6 m)**	**0.003**	**0.595**	**0.423–0.835**	**0.036**		**0.686**	**0.482–0.976**
**Third-line treatment** **(Anti-angiogenic drugs vs. Chemotherapy ± targeted drugs)**	**0.001**	**1.863**	**1.284–2.703**	**0.000**		**2.203**	**1.514–3.206**
Third-line chemotherapy (Re-challenge vs. New regimens)	0.790	1.051	0.730–1.512	0.355		1.208	0.809–1.803
Third-line targeted drugs (Re-challenge vs. New drugs)	0.174	1.308	0.888–1.926	0.146		1.359	0.898–2.055
Previous use of anti-angiogenic therapy (Yes vs. No)	0.925	1.021	0.666–1.564	0.584		0.882	0.564–1.381
**Later-line treatment (Yes vs. No)**	-	-	-	**0.000**		**0.496**	**0.349–0.703**
***Cox multivariate analyses***							
Resection of primary tumor							
Palliative vs. Radical resection	0.977	0.995	0.697–1.419	0.728		1.073	0.722–1.594
None vs. Radical resection	0.062	1.539	0.978–2.421	0.277		1.310	0.805–2.132
**RAS/RAF status** **(Wild-type vs. Mutant-type)**	-	-	-	**0.021**		**0.613**	**0.405–0.929**
**PFS of front-line treatment** **(PFS1 + PFS2 > 10.6 m vs. ≤10.6 m)**	**0.036**	**0.685**	**0.480–0.976**	**0.030**		**0.670**	**0.467–0.963**
**Third-line treatment** **(Anti-angiogenic drugs vs. Chemotherapy ± targeted drugs)**	**0.029**	**1.598**	**1.049–2.435**	**0.001**		**2.004**	**1.313–3.059**
**Later-line treatment (Yes vs. No)**	-	-	-	**0.004**		**0.564**	**0.380–0.836**

*HR: Hazard ratio, CI: confidence interval, PFS: Progression-free survival, OS: Overall survival*

Furthermore, since our sample size was relatively limited, we conducted a post hoc (a posteriori) power analysis. The power was calculated as 0.90 for PFS analysis and 0.95 for OS analysis, respectively, suggesting the high credibility of our results.

## Discussion

Our study evaluated the efficacy of chemotherapy in the third-line setting of mCRC. Results from this study indicate that more than 60% of mCRC patients may experience clinical benefit from third-line chemotherapy despite prior exposure to irinotecan or oxaliplatin. Compared to anti-angiogenic monotherapy, chemotherapy seems to have more survival advantages in ‘selected’ patients, including those with RAS/RAF mutant tumors, or with longer PFS (more than 10.6 m) of front-line therapy, or with larger tumor burden.

For mCRC, there are universally acknowledged first-line and second-line treatment regimens. Our study showed that the switch of oxaliplatin-based regimen in first-line to irinotecan-based scheme in second-line was the most frequently used strategy (Fig. 1), which is accordance with previous investigations [20]. With regard to the third-line treatment for mCRC, regorafenib, TAS-102 and cetuximab in combination with irinotecan are recommended, however, there is no consensus on who is more suitable for particular strategy. Clinicians will determine individualized third-line therapy based on molecular characteristics, previous used regimens, residual toxicity, accessible drugs and so on. In a real-world study of mCRC in Australia, the majority of patients chose chemotherapy as their third-line therapy and more than four-fifths of them were given as chemotherapy rechallenge [21]. Similarly, more mCRC patients in our study chose third-line chemotherapy (78.1%) and the re-challenge rate was 65%.

As a frequently applicated third-line option, the efficacy of chemotherapy has been discussed in previous studies. A Japanese study demonstrated that chemotherapy rechallenge was a valuable option [22]. The clinical benefit rate of oxaliplatin or irinotecan re-challenge was reported to be 75.5% in an American cohort [23]. In another Italy RETROX-CRC retrospective study, oxaliplatin retreatment produced further response rate in around one-fifth of patients [24]. The results are controversial as to the comparison of the efficacy of chemotherapy to that of anti-angiogenic monotherapy. A Chinese study containing 105 mCRC patients who failed at least two lines of chemotherapy concluded that anti-angiogenic monotherapy was superior to chemotherapy [25]. Conversely, a Japanese retrospective study showed that chemotherapy exerted more survival benefit than regorafenib. Patients treated with TAS-102 had better tumor response than those treated with regorafenib [26], illustrating the superiority of chemotherapy in third-line setting. Our study also confirmed the superiority of third-line chemotherapy compared to anti-angiogenic monotherapy. Although the chemotherapy regimens in our study included rechallenge scheme and new drugs, nearly two-thirds of patients in the latter group were prescribed traditional oxaliplatin or irinotecan, suggesting that chemotherapy reintroduction is a valuable option and will leave more effective drugs for the later-line therapy. Moreover, we found that the combination of chemotherapy with bevacizumab in third-line setting did not further increase the efficacy, which might be influenced by the limited sample size of our study. However, it should be noted that although the SUNLIGHT trial showed that the addition of bevacizumab to third-line treatment may prolong survival in heavily pretreated mCRC patients [8], the OS benefit observed in SUNLIGHT trial is larger than the magnitude of benefit observed in other bevacizumab-based combination studies. Thus, the clinical value of bevacizumab in the third-line setting of mCRC patients, especially its combination with traditional chemotherapy drugs in third-line needs more exploration.

The biomarkers to predict response to drugs currently used in the later-line treatment are truly unmet clinical needs, especially because patients may have serious and persisting side effects in this period [27–29]. Accurate patient selection improves the therapeutic efficacy of any regimen. APC mutation and FGFR1 amplification were reported to be associated with the efficacy of regorafenib [30]. Additional examination of RAS/RAF status could contribute to the selection of mCRC patients who might be likely to benefit from third-line anti-EGFR agents [31]. Because RAS/RAF mutant tumors are often accompanied by aggressive characteristics, such patients need more powerful treatment options. In our study, the OS of patients in the anti-angiogenic treatment group reached only 5.2 m, which was lower than the data of clinical trials, mainly due to the high proportion of RAS/RAF mutant type (80%) in this group. However, we also found that in patients with RAS/RAF mutation, the efficacy of third-line chemotherapy was superior to that of anti-angiogenic monotherapy, whereas the difference disappeared in the RAS/RAF wild population, indicating the predictive value of RAS/RAF status in the decision-making of third-line chemotherapy.

In addition to molecular biomarkers, clinical selection criteria are also frequently used strategies due to its simplicity. In ovarian cancer, the most important predictor of response to carboplatin reintroduction is the length of PFS between different lines of treatments [32, 33]. In mCRC, the earliest information about the potential role of the oxaliplatin-free interval as a predictive marker came from the FOLFOX stop-and-go approach. In a pooled analysis exploring FOLFOX reintroduction, both response rate and PFS nearly doubled when the oxaliplatin-free interval was more than 6 months [34]. Researchers failed to reconstruct the oxaliplatin-free interval from the RETROX-CRC study, however, they found that the trend of better response to oxaliplatin retreatment in patients who used oxaliplatin as adjuvant therapy [24], which indicated longer time interval in some degree. In addition, chemotherapy rechallenge was more efficient in patients who achieved PR or SD in front-line therapies [22]. The above studies have confirmed that effective front-line chemotherapy is the prerequisite for the benefit from third-line chemotherapy. Thus, we calculated the cut-off value of PFS of front-line chemotherapy. As expected, patients with longer PFS of front-line therapy can benefit more from chemotherapy than anti-angiogenic monotherapy.

Our study has several limitations. Firstly, the sample size of this study is relatively limited. We retrospectively collected as many patients as possible who had available third-line treatment data and accessible follow-up information at our center, but due to the high lost follow-up rate in the retrospective study, we ultimately obtained treatment data and survival outcomes from only 183 patients. Because of many patients undergoing outpatient visits and treatment, the collection of adverse effect information was imperfect and these data were not included in the final analysis. According to incomplete statistics, in the chemotherapy group, more than half of patients experienced leukopenia or neutropenia, and gastrointestinal reactions such as nausea and vomiting occurred in nearly 20% of cases, whereas the most common adverse effects in the anti-angiogenic monotherapy group were hand-foot skin reaction, followed by hypertension. Considering the heterogeneity of treatment, it is necessary to enroll more patients from different centers or conduct prospective controlled studies to further validate our conclusions in future. Secondly, since we collected consecutive cases, some patients (10/143, 7%) in the chemotherapy group received chemotherapeutic drugs unapproved in guidelines. Under specific circumstances that there was no standard third-line treatment or medication was not accessible, patients participated in clinical trials on third-line chemotherapy, such as gemcitabine (we previously found the potential therapeutic effect of gemcitabine in mCRC [35] and tried to validate it in more patients). Although it was not recommended in the guidelines, this group of patients accounted for a relatively small proportion and had limited impact on the final results. Thirdly, we were unable to perform a comprehensive comparison between new chemotherapeutic drugs and rechallenge strategies because of the relatively small sample size, which would have impaired the statistical soundness of the analysis. Larger multi-center real-world analyses or prospective randomized trails are needed to further consolidate our findings.

## Conclusions

To conclude, we found that third-line chemotherapy was effective, and compared to anti-angiogenic monotherapy, chemotherapy might be the better choice for ‘selected’ mCRC patients. Those with more aggressive characteristics (RAS/RAF mutant, larger tumor burden) or better efficacy of previous chemotherapy (longer PFS of front-line therapy) were more appropriate for third-line chemotherapy. This study has certain significance in the clinical decision-making of third-line therapy for mCRC, and further translational studies, to identify molecular biomarkers linking the tumor biology to chemotherapy sensitivity, are warranted.

### Electronic supplementary material

Below is the link to the electronic supplementary material.

Supplementary Material 1

Supplementary Material 2

Supplementary Material 3

Supplementary Material 4

Supplementary Material 5

Supplementary Material 6

## Data Availability

The datasets used and/or analyzed during the current study are available from the corresponding author on reasonable request.
