# Supplementary material for: Chemotherapy re-use versus anti-angiogenic monotherapy as the third-line treatment of patients with metastatic colorectal cancer: a real-world cohort study
Source: BMC Cancer. 2024 Mar 5;24:302. doi: 10.1186/s12885-024-12072-5 (PMC10916076; doi:10.1186/s12885-024-12072-5)
Supplement: Supplementary file 1 — Supplementary Material 1 [file 12885_2024_12072_MOESM1_ESM.docx]

**Supplementary figure legends**

**Figure S1. The front-line treatment patterns of mCRC patients in our cohort. (A)** The first-line treatment patterns. **(B)** The second-line treatment patterns.

**Figure S2. The third-line treatment patterns of mCRC patients in our cohort. (A)** Metastatic sites at third-line treatment. **(B)** The third-line treatment patterns.

**Figure S3. Survival curves of mCRC patients according to third-line chemotherapy regimens. (A)** The progression-free survival curves of patients stratified by third-line chemotherapy. **(B)** The overall survival curves of patients stratified by third-line chemotherapy. PFS: progression-free survival. OS: overall survival.

**Figure S4. The overall survival curves of all patients according to later-line treatment.** OS: Overall survival.

**Figure S5. ROC curve.** AUC: Area under curve.
