# Supplementary figures and images for: Chemotherapy re-use versus anti-angiogenic monotherapy as the third-line treatment of patients with metastatic colorectal cancer: a real-world cohort study
Source: BMC Cancer. 2024 Mar 5;24:302. doi: 10.1186/s12885-024-12072-5 (PMC10916076; doi:10.1186/s12885-024-12072-5)

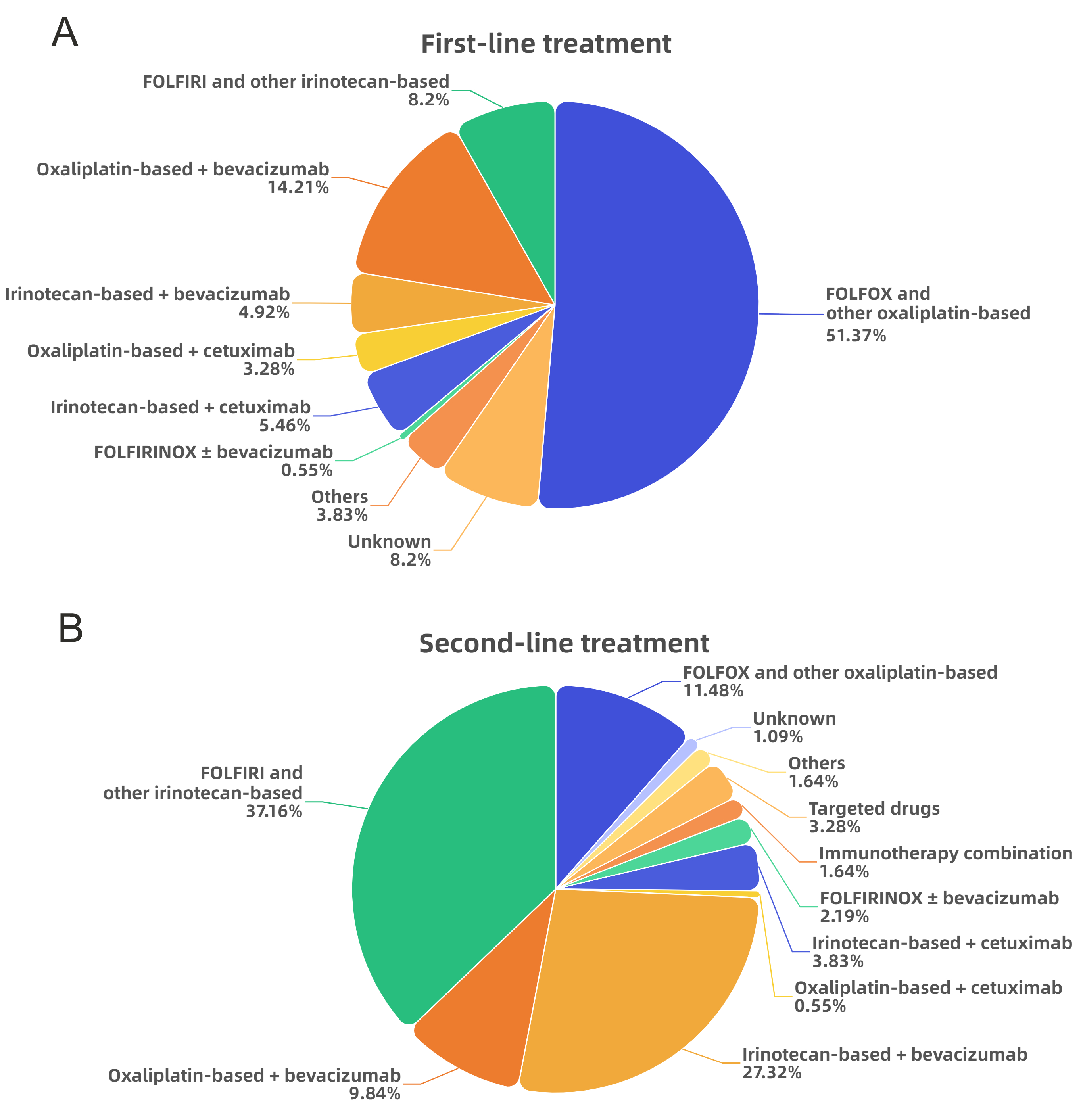

Supplement: Supplementary file 2 — Supplementary Material 2 [file 12885_2024_12072_MOESM2_ESM.tif]

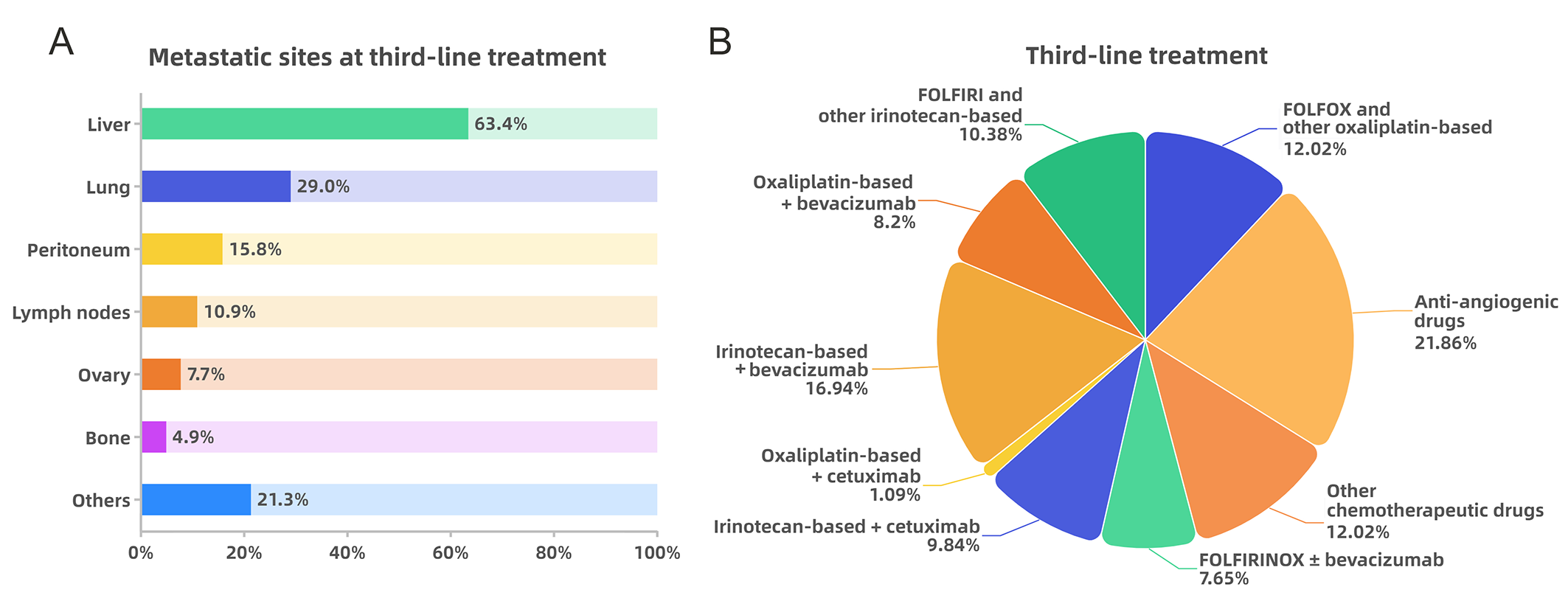

Supplement: Supplementary file 3 — Supplementary Material 3 [file 12885_2024_12072_MOESM3_ESM.tif]

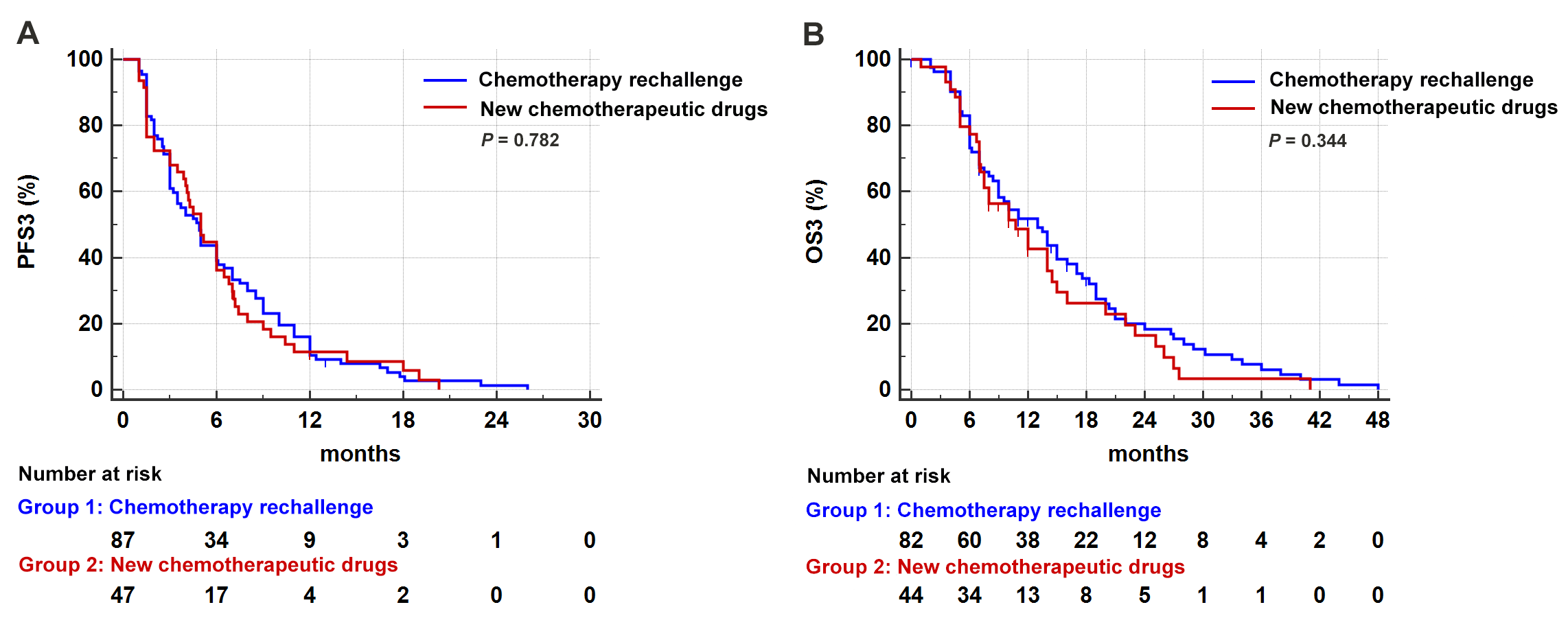

Supplement: Supplementary file 4 — Supplementary Material 4 [file 12885_2024_12072_MOESM4_ESM.tif]

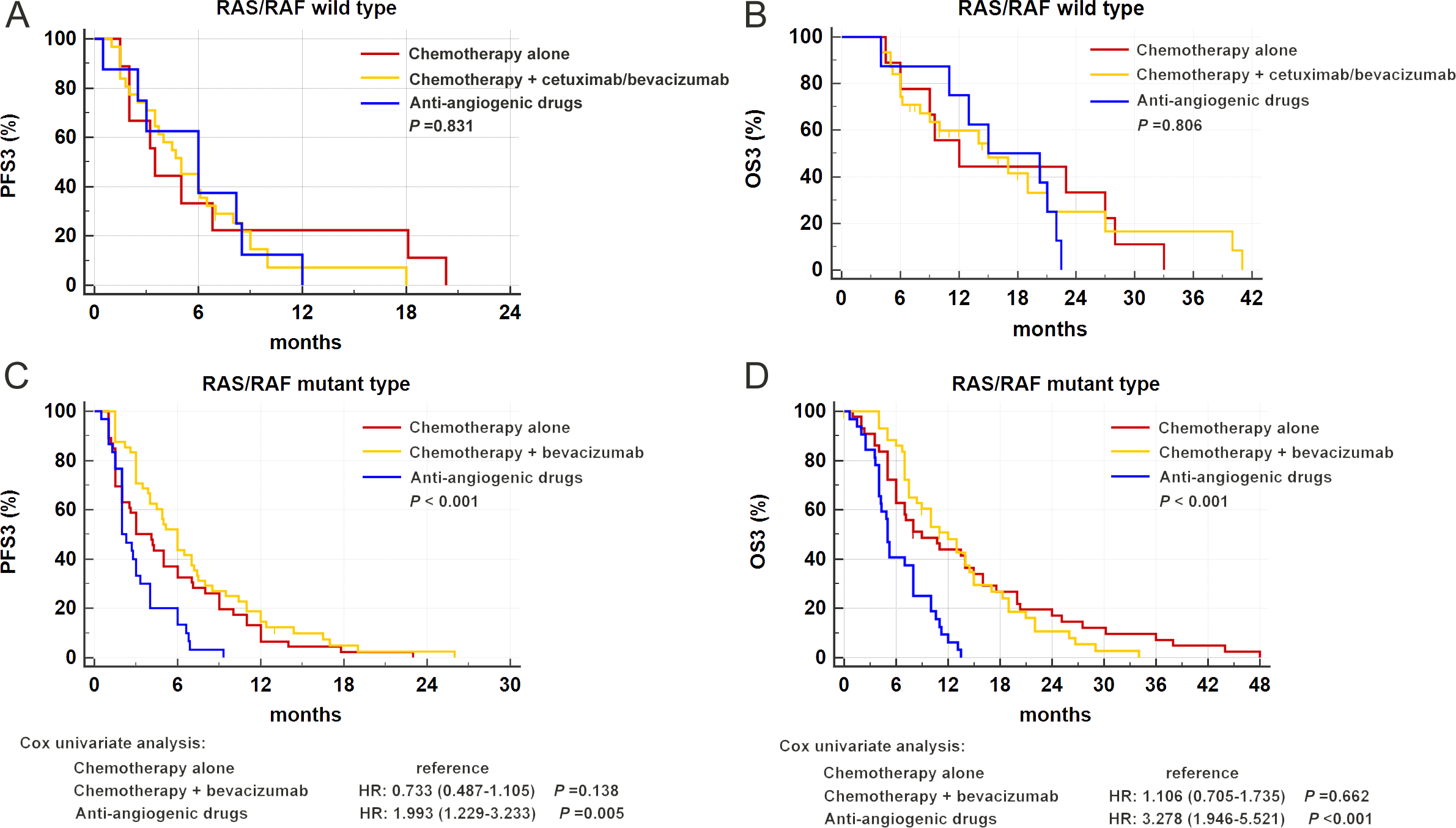

Supplement: Supplementary file 5 — Supplementary Material 5 [file 12885_2024_12072_MOESM5_ESM.tif]

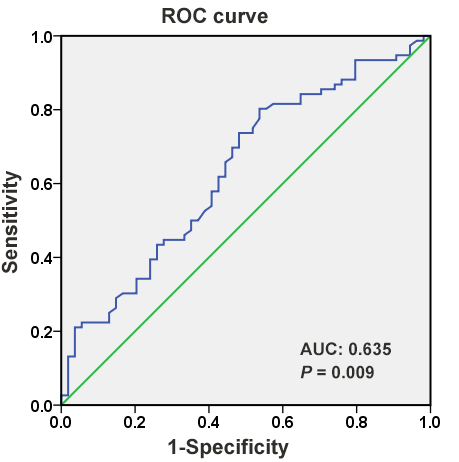

Supplement: Supplementary file 6 — Supplementary Material 6 [file 12885_2024_12072_MOESM6_ESM.tif]
